# Bilateral Breast Masses with a Rare Etiology

**DOI:** 10.1155/2013/412368

**Published:** 2013-08-28

**Authors:** Friederike Thieringer, Gideon Sartorius, Katrin Kalf, Viola Heinzelmann, Marcus Vetter

**Affiliations:** ^1^Department of Gynecology and Obstetrics, University Hospital Basel, 4031 Basel, Switzerland; ^2^Clinic of Gynecological Endocrinology and Reproductive Medicine, University Hospital Basel, 4031 Basel, Switzerland; ^3^Department of Gynecologic Oncology, University Hospital Basel, 4031 Basel, Switzerland; ^4^Department of Medical Oncology, University Hospital Basel, 4031 Basel, Switzerland

## Abstract

Breast masses have a variety of benign and malignant etiologies. We present the case of a 28-year-old woman with bilateral large painful breast masses that developed rapidly in the three weeks before first presentation. Further investigation revealed bilateral ovarian masses. Biopsies of both ovarian masses were taken, and the pathology reported Burkitt's lymphoma. Additional staging with a PET scan was suggestive of bone marrow involvement, but bone marrow biopsy was negative. Examination of the cerebrospinal fluid did not identify malignant cells. The patient underwent CODOX-M/IVAC chemotherapy, and a complete response was demonstrated after one cycle of treatment. Six months after finishing chemotherapy the patient remained in complete remission. To our knowledge this is the first case reporting simultaneous involvement of breast, ovaries, and bones in Burkitt's lymphoma. Gynecologists and oncologists should be aware of this pattern. Polychemotherapy treatment must be initiated rapidly with curative intent.

## 1. Introduction 

Bilateral breast masses have a variety of etiologies, for example, sarcoma [1], fibromatosis [2], carcinoma [3], or lymphoma [4]. A rapid diagnostic referral is warranted because of possible malignancy. We present the case of a 28-year-old woman who presented with bilateral breast masses. She was found also to have bilateral ovarian masses. Further investigation demonstrated Burkitt's lymphoma. This high grade lymphoma was first described by Burkitt in 1958 in African children [5] and has the most rapid proliferation rate of all known tumours. However, polychemotherapy can result in cure.

## 2. Case

**Figure 1:**
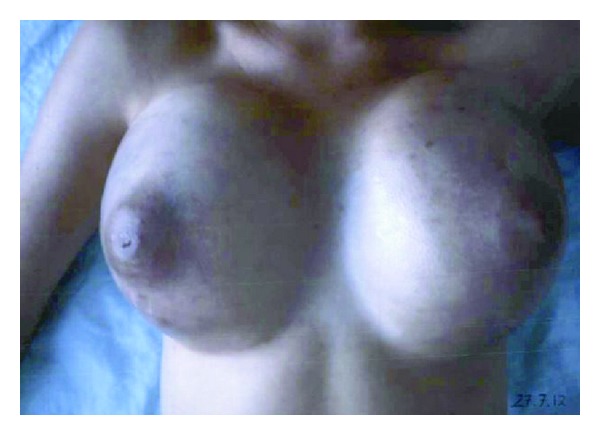
Initial clinical presentation, massive enlarged bilateral breast masses.

**Figure 2:**
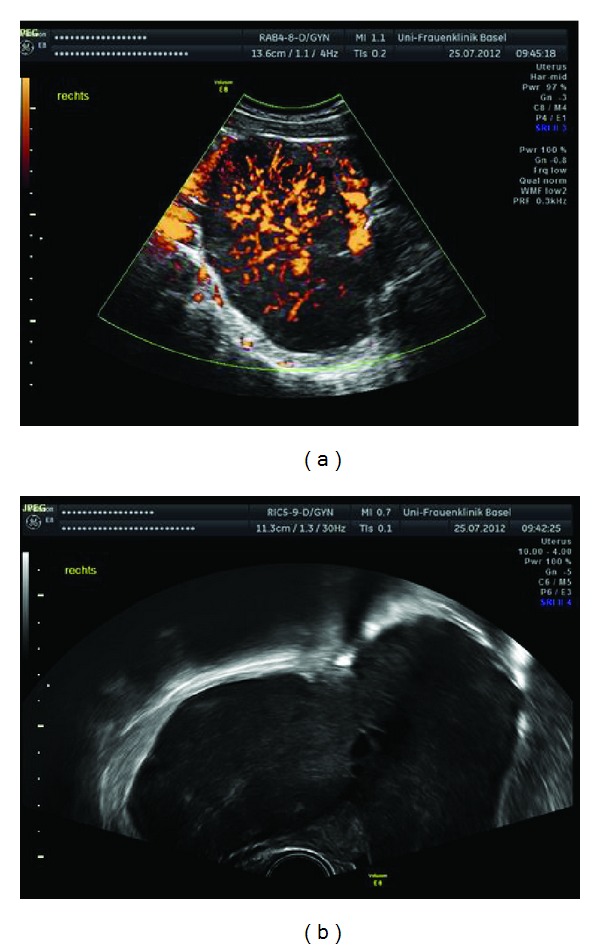
Initial ovarian ultrasound showed bilateral ovary masses.

A 28-year-old Caucasian woman, G0, was referred to our hospital because of obvious bilateral breast masses that had grown rapidly within three weeks. Clinical examination demonstrated gross swelling of both breasts with bluish skin colour alteration due to contusion (Figure 1). Further gynaecological investigation revealed bilateral ovarian masses measuring up to 11 cm on the right side and 8 cm on the left (Figure 2). No ascites was present. Initial thoraco-abdominal CT scan confirmed the pelvic tumors, but no further evidence of disease. 

Blood tests revealed a normal hemoglobin of 127 g/L, normal white blood cell count, and a platelet count, of 194 × 10^9^/L. The serum chemistry profile showed uric acid 515 *μ*mol/L (abnormal high), lactic dehydrogenase 487 U/L (abnormal high), and CRP 17.5 mg/L (abnormal high). Her hormonal status, including TSH, FSH, LH, oestradiol, Prolactin, DHEA, testosteron, SHBG, AFP, and insulin, was normal. The tumor markers CEA and CA-125 were also normal. Cytological evaluation by fine needle aspiration of the breast was not conclusive.

Assuming a possible hormonal cause for the massive breast tumors, a trial of mifepristone treatment (200 mg) was undertaken—this is an antiprogesterone therapy given with the aim of inhibiting the proliferation of breast tissue. No effect was demonstrated within 12 hours.

Finally, diagnostic laparoscopy showed a further increase in size of the ovarian tumors compared to the initial ultrasound scan just two days before. Histology (from a biopsy taken from both ovaries) revealed high grade Burkitt's lymphoma. 

Viral serologies including hepatitis A/B/C and HIV were all normal. EBV serology indicated previous infection (EBV EBA (IgG) AK 482.0 E/mL, EBV VCA IgG 407.0 U/L).

**Figure 3:**
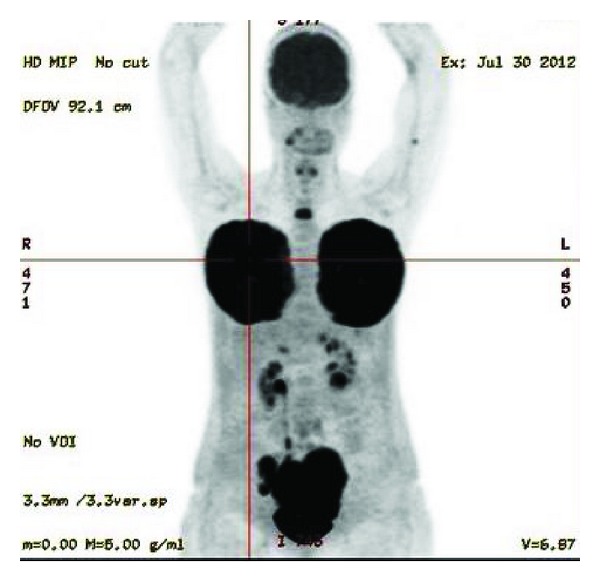
Initial PET scan showed massive FDG activation in bilateral breast masses and ovaries.

Further investigation with a PET scan was suggestive of diffuse skeletal involvement, but bone marrow aspiration and histology were negative. It showed massive FDG activation in bilateral breast masses and ovaries (Figure 3). Cranial MRI and lumbar puncture excluded central nervous system (CNS) involvement. 

Prephase treatment was initiated with 100 mg of oral prednisolone. The patient had a rapid improvement in her symptoms within 12 hours. This was followed by treatment with systemic intensive polychemotherapy according to the CODOX-M/IVAC protocol [6]. Rituximab therapy was also included at the beginning of each cycle (day 0). A total of 4 cycles were administered without major toxicities (mucositis, abdominal pain, and constipation; all grade one or two).

**Figure 4:**
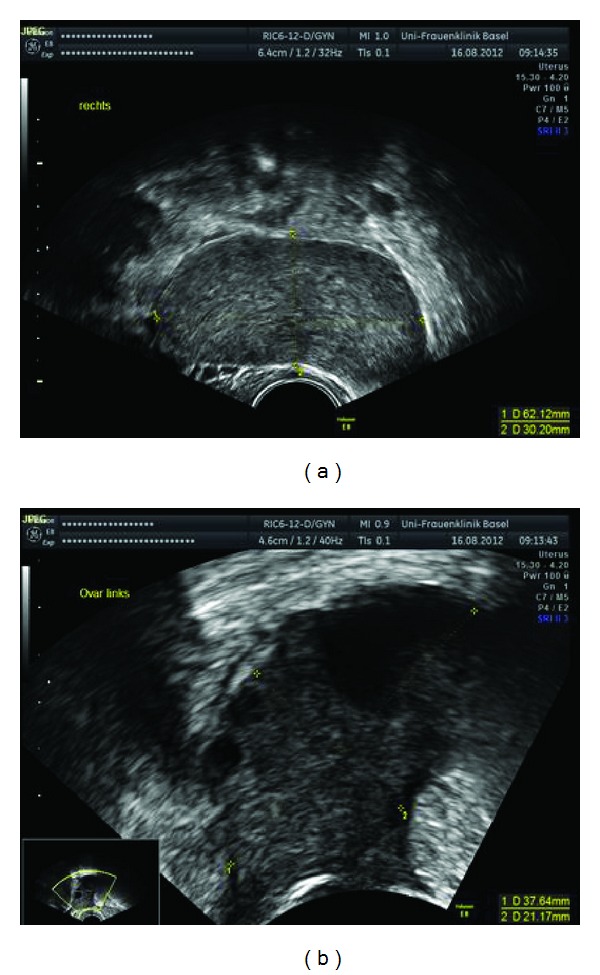
Ovarian ultrasound after 1st cycle of chemotherapy.

After one cycle of chemotherapy, the breast volume reduced almost to normal, as did the ovaries (Figure 4). A CT scan after 2 cycles of treatment demonstrated complete remission. At the time of writing, six months after completing treatment, the patient is in complete remission.

## 3. Discussion

Burkitt's lymphoma was first described in 1958 by Denis Parsons Burkitt. There are three different types of Burkitt's lymphoma at presentation: sporadic, most common in Europe and the US, endemic, and HIV-associated Burkitt's lymphoma, both of which are most common in Africa. Burkitt's lymphoma is an aggressive B-cell non-Hodgkin lymphoma and typically presents with large abdominal masses [7, 8]. Less than 1% of patients with malignant lymphoma initially present with ovarian tumors and a primary malignant lymphoma of the breast is a very rare diagnosis with an incidence of only 0,05–0,25% of all malignant breast neoplasms [8, 9].

To our knowledge this is the first case with simultaneous involvement of breast, ovaries, and possibly bones. The presentation with painless swelling of both breasts suggested an endocrine cause. Further investigation showed enlarged ovaries with no other abdominal masses. There were no specific findings in blood tests (tumor marker, hormones, blood count, serum chemistry profile, etc.) or cytological examination of the breast. A variety of differential diagnoses were considered, including neuroendocrine and germ cell tumours of the ovary, but the patient's rapid clinical deterioration with threatened ulceration of the breasts required an urgent diagnosis. Thus, before the results of hormonal tests and the trial of antiprogesterone treatment were available, laparoscopy was undertaken for a histological sampling, establishing the diagnosis of a malignant lymphoma. 

Most of the reported cases of Burkitt's lymphoma underwent surgery, but debulking of the tumor does not appear to influence clinical outcome [8, 10]. Extensive abdominal surgery is potentially very morbid and can have a significant impact on the patient's performance status. In addition, a treatment delay due to convalescence after surgery is not justifiable in this highly chemosensitive disease. Burkitt's lymphoma is a systemic disease with a proliferation index of nearly 100% and a cell doubling time of approximately 25 hours. It is very sensitive to chemotherapy, so an intensive polychemotherapy regimen that includes rituximab is considered to be the treatment of choice [10, 11], with a 3-year survival rate of up to 90% [12]. High-dose chemotherapy and stem cell transplantation an option but not established as a first-line standard. 

## 4. Conclusion

In patients with rapidly growing breast masses, lymphoma, in particular Burkitt's lymphoma and other aggressive B-cell lymphomas, should be considered in the differential diagnosis.

We recommend transfer of the patient into a specialist cancer centre, where interdisciplinary collaboration as well as diagnostic resources (i.e., PET, MRI, and Pathology) is available. A rapid and reliable histological diagnosis is critical, enabling urgent treatment with chemotherapy. This diagnosis carries a high risk of recurrence and should be aggressively treated by intensive polychemotherapy. A standard option is the CODOX-M/IVAC Protocol.
